# Reference Ranges of Selenium in Plasma and Whole Blood for Child-Bearing-Aged Women in China

**DOI:** 10.3390/ijerph19084908

**Published:** 2022-04-18

**Authors:** Yang Cao, Huidi Zhang, Jingxin Yang, Qingqing Man, Pengkun Song, Deqian Mao, Jiaxi Lu, Lichen Yang

**Affiliations:** Key Laboratory of Trace Element Nutrition of National Health Committee, National Institute for Nutrition and Health, Chinese Center for Disease Control and Prevention, Beijing 100050, China; yasmine0814@163.com (Y.C.); zhanghuidi1114@126.com (H.Z.); jingxinyang2022@126.com (J.Y.); manqq@ninh.chinacdc.cn (Q.M.); songpk@ninh.chinacdc.cn (P.S.); maodq@ninh.chinacdc.cn (D.M.); lujx@ninh.chinacdc.cn (J.L.)

**Keywords:** reference range, blood Se, child-bearing-aged women

## Abstract

Selenium (Se) is a “dual-surface” element. Both Se-deficiency and Se-overload have bad effects on humans. The amount of Se in the blood is a good indicator of Se intake, and there are considerable differences in the reference ranges among different regions and populations. The purpose of this study was to establish the age-specific reference interval of blood Se in healthy child-bearing-aged women in China. A total of 187 healthy women aged 18–45 years old were enrolled with strict inclusion criteria from the China Adult Chronic Disease and Nutrition Surveillance (2015 CACDNS) database to establish the reference interval of Se. Plasma and whole-blood Se were detected by inductively coupled plasma mass spectrometry (ICP-MS). The reference range (RR) estimated as P_2.5_–P_97.5_ percentiles (geometric mean) was 73.81–140.75 (100.94) μg/L and P_2.5_–P_97.5_ percentiles (median) 81.06–164.75 (121.05) μg/L for plasma and whole-blood Se, respectively. The proposed RR of plasma Se in this study was used to evaluate the Se nutritional status of a representative sample of 1950 women of child-bearing age who were randomly selected from 2015 CACDNS. The proportion of Se level lower than P_2.5_ cut-off value was 24.05%, and there were 5.08% child-bearing-aged women with plasma Se higher than the upper limit of RR. Women in the western and rural areas tend to have lower Se levels.

## 1. Introduction

Selenium (Se) is an essential trace element that is ubiquitous in nature. The nutritional status of Se in human organisms varies depending on Se intake, gender, age, bioavailability, and other factors [1,2]. It was confirmed that severe inadequate Se intake was closely related with Keshan Disease (KD) and Kashin–Beck Disease (KBD). Low Se status has been associated with increased risk of mortality, poor immune function, and cognitive decline. However, Se is also a kind of “dual-surface” element. Excessive Se can cause selenosis [3] and other related health damage [4]. Nowadays, except for the typical diseases cased by Se shortage and overdose, a growing number of studies show that Se deficiency and excessive Se were associated with many chronic diseases, such as diabetes, heart disease, autoimmune diseases, and certain types of cancers [5]. Thus, it is important to assess the nutritional status of Se accurately.

Since there are both Se-deficient and Se-toxic regions in China [6], it was not recommended to assess Se intake by Food Composition Table due to the big differences in Se content in local soils or foods. Total Se in blood is a good indicator for Se status assessment, which is more sensitive to reflecting the changes of human Se intake and storage. Whole-blood Se is used to reflect long-term Se absorption and metabolism; plasma or serum Se are usually used to evaluate short-term Se status or intake [7]. Because serum Se concentration is usually identical to that of plasma, plasma is used in this study.

Currently, many countries have established the reference range of blood Se based on their population data, including the USA [8], Belgium [9], Queensland [10], Korea [11], Japan [12], etc. Some of these studies found similar blood Se between men and women, while some reported higher Se in men [13,14,15,16]. It is inappropriate to extrapolate Se-related findings from studies conducted with one sex to the other [1]. In addition, child-bearing age is a very important stage for women, which may affect their nutritional status when they in pregnancy and the health of next generation [17,18].

There are also some suggested reference ranges in different regions in China [19,20,21,22,23], while these data differ greatly from each other. Thus, the current study aims to establish the age-specific reference interval of blood Se in Chinese healthy child-bearing-aged women based on the China Adult Chronic Disease and Nutrition Surveillance (2015) database. The result is of great importance not only for human biomonitoring, but for assessment of the clinical health effect caused by deficiency or overload of Se.

## 2. Materials and Methods

### 2.1. Subjects

A total of 1950 women of child-bearing age (18–44 years) were selected by using multi-stage stratified sampling and population ratified random sampling method from the China Adult Chronic Disease and Nutrition Surveillance (2015) database, which is a national representative cross-sectional survey, taking into account regional types and age subgroups [24]. Then, we enrolled 187 healthy child-bearing-aged women with strict inclusion and exclusion criteria described from 1950 women of child-bearing age in a previous study [25]. The BMI, blood lipid, blood pressure and blood glucose, etc., of all the individuals were within the normal range. This project was authorized by the Ethics Committee of the National CDC Institute of Nutrition and Health, China CDC (file No.: 201519-a). The study was conducted in accordance with the Declaration of Helsinki, and informed consent was obtained from all the included patients.

### 2.2. Data Collection

The physical examination was carried out by trained medical personnel in accordance with standard procedures. For the measurement of height, weight, waist circumference and blood pressure, the details can be found in our published work [26]. Blood collections were collected from the forearm vein of fasting individuals using steel needles, into the vacuum EDTA-K2 anticoagulant blood collection vessel.

### 2.3. Determination of Plasma and Whole-Blood Se Concentration

Plasma samples were diluted with 0.5% (*v*/*v*) HNO3 (1:20). Whole bloods were diluted with 0.5% (*v*/*v*) HNO3 and 0.05% (*v*/*v*) Triton-X-100 (1:25). In order to avoid contamination of samples, the experiment was carried out on a clean bench. Trace elements dissolution was avoided with 15 mL centrifuge tubes (Corning, NY, USA). Se concentration in the plasma and whole blood was measured by inductively coupled plasma mass spectrometry (ICP-MS, PerkinElmer, NexION 350, Waltham, MA, USA). The plasma quality-control samples (Clincheck, serum Level-2, Munich, Germany; Seronorm, serum Level-2, Billingstad, Norway) were used to monitor and analyze at intervals of 20 samples to ensure the precision and accuracy of detection. The whole-blood quality-control samples (Clincheck, whole blood Level-2, Munich, Germany; Seronorm, whole blood Level-2, Billingstad, Norway) were used to monitor and analyze at intervals of 10 samples.

The rate of recovery of Se was 105.87% and 110.53% in plasma and whole blood, respectively. The inter-day precision of Se for plasma and whole blood was 4.86% and 6.52%, while the intra-day precision values were 5.83% and 8.01%. The limit of detection (LOD) was 0.38 μg/L and 0.75 μg/L for plasma and whole-blood Se. The limit of quantitation (LOQ) was 1.26 μg/L and 2.51 μg/L for plasma and whole-blood Se.

### 2.4. Statistical Analysis

To estimate reliable reference values, the International Union of Clinical Chemistry (IFCC) recommends at least 120 observations [27]. The data analysis was performed using IBM SPSS version 26.0 software (SPSS Inc., Chicago, IL, USA). The result for general characteristics was presented by GM, median, P_25_ and P_75_. The reference range was calculated as lower limit and upper limit of 95% confidence intervals (CI). In this study, the concentration of plasma Se was non-normally distributed and whole-blood Se was normally distributed. Therefore, the result of Se levels was shown by GM, P_2.5_ and P_97.5_ for plasma, P_2.5_ and P_97.5_ for whole blood, respectively. The Kruskal–Wallis Test was used for analysis of concentration of plasma Se differences between groups (age, area, and residences). The differences of whole-blood Se between the two groups were tested by Student’s *t* test and more than two groups were tested by one-way ANOVA. Statistical difference was considered when *p* < 0.05. Statistical difference was considered when *p* < 0.05. The concentration of plasma Se of the random sample of 1950 child-bearing-aged women were non-normally distributed. The results of plasma Se levels were showed by geometric mean (Geom. Mean) and detected with a Kruskal–Wallis Test and a chi-square test.

## 3. Results

### 3.1. Population Characteristics

This study included 187 healthy child-bearing-aged women between the ages of 18–45 years. Description of the general characteristics were presented in Table 1. All the basic demographics and the related clinical parameters were in the normal ranges and met our inclusion and exclusion criteria.

### 3.2. Plasma and Whole Blood Concentrations of Se

As shown in Table 2, the reference range of plasma Se and whole-blood Se were 73.81 to 140.75 μg/L and 81.06 to 164.72 μg/L, respectively. There were a few subjects with extremely low values. No significant differences of plasma Se were found among different age-groups and areas, but the median plasma Se concentration in urbans was higher than that in the rural region. Whole-blood Se concentration was higher in the eastern area than that in the midlands and western area, but no significant difference was observed among age subgroups and residence types.

### 3.3. Reference Ranges of Plasma/Serum and Whole-Blood Se in Various Countries or Regions

Table 3 showed the reference range of plasma/serum and whole-blood Se established by different countries and regions for their own populations. Belgium [9], Queensland [10], and Serbia [13] proposed both the reference range of plasma/serum and whole blood Se. Korea [11], Taiwan [14], Japan [12], Beijing [19], and Jinan [20] in China only developed the related plasma or serum Se reference range. Southern Brazil [16], USA [8], Italy [15] and Shannxi [21], Chengde [22], Hunan [23] in China reported the related reference range of whole blood Se. Except for Serbia [13] and Jinan [20], the reference ranges of plasma/serum Se from Belgium [9], Queensland [10], Korea [11], Taiwan [14], Japan [12], and Beijing [19] are all at the same order of magnitude as the current study. However, the concentration of whole-blood Se varies greatly among different countries.

### 3.4. Distribution of Plasma Se in the National Representative Child-Bearing-Aged Women in China

A total of 1950 child-bearing-aged women’s plasma Se distribution by age group, areas and residences variables are presented in Table 4. According to the current result of plasma Se reference ranges, the difference was significant between east, midland and western areas. Moreover, the difference in both urban and rural plasma Se concentration was statistically significant. The prevalence of plasma Se lower than the P_2.5_ cut-off level was 24.95%, and the proportion of plasma Se higher than the P_97.5_ threshold was 5.08%.

More child-bearing-aged women in the western region and rural areas has a plasma Se level below the P_2.5_ cut-off of the reference range.

## 4. Discussion

Most countries established reference intervals of kinds of trace elements. Nowadays, there are no universal reference ranges or cut-off values for total Se in the blood [28]. The 95% confidence interval (95% CI) method is usually used to establish the population reference interval. As mentioned above, the range of plasma Se in Belgian [9], Queensland adult [10], Serbian [13], and whole-blood Se in American adults [8] used a 95% CI method to describe the distribution of Se. This method, usually based on national nutritional monitoring data, contributes to a better understanding of the overall distribution of the region. However, this method cannot exclude some diseases that influence Se levels, such as diabetes mellitus, metabolism syndrome, and so forth [29,30]. Genetic and geographic differences can influence the blood Se level [13,31]. It is recommended that countries or regions should establish a reference range based on their own population characteristics and dietary pattern. Therefore, strict inclusion and exclusion criteria were used in our study to define a “healthy” reference population for child-bearing-aged women.

In this study, the reference range of plasma Se in Chinese healthy child-bearing-aged women was 73.81 to 140.75 μg/L. The result was close to Korea [11] (P_2.5_–P_97.5_ 79.07–166.46 μg/L). A total of 258 samples was reported in Korea [11], so it seems that the sample size was higher than our study. However, the population distribution age was 12–78 years old and covered both genders in the Korean study [11]. In contract, our study was more age-specific and just focused on the child-bearing-aged women, so the sample size was quite comparable with other studies. Belgium [9] (P_2.5_–P_97.5_ 61.6–122 μg/L), Queensland [10] (P_2.5_–P_97.5_ 98–160 μg/L), and Serbia [13] (P_2.5_–P_97.5_ 37.4–97.5 μg/L) also used the 95% CI method to estimate the plasma/serum Se reference range of adults in their countries, and the data of Belgium [9] and Queensland [10] (P_2.5_–P_97.5_ 98–160 μg/L) were closed to the results of this study. The result of Serbian [13] study was lower than the current study and included two genders. Compared with other Asian regions, the reference range of Japan [12] (min–max 76.9–149.4 μg/L) and Taiwan [14] (min–max 40.5–186 μg/L) was reported earlier and presented in the form of “min–max”. Although the traditional AAS method was used in these studies, good consistency can be found with our results (min–max 64.88–145.22 μg/L). Among them, Taiwan [14] used the distribution of 2755 general adults over 15 years, so the distribution interval was broader than in the present study. It further proved that the establishment of a “healthy population” can help to obtain a more accurate distribution range. There are also some studies on the range of plasma/serum Se for normal population in some regions of China, such as Beijing [19] (min–max 35.2–160.4 μg/L) and Jinan of Shandong province [20] (P_2.5_–P_97.5_ 35.58–85.96 μg/L) for women. The results of Beijing [19] were closed to the results of this study, and Jinan [20] was lower than the study. As shown in Table 2, the sample distribution of our study covers a wider geographical and urban–rural distribution. It was reported that the areas and residences distribution will impact on Se levels [32,33].

The Se concentration in whole blood was generally higher than that in plasma. The whole-blood Se reference range was 81.06–164.72 μg/L in this study. This result was quite a bit lower than the U.S. [8] (P_2.5_–P_97.5_ 152.3–251.3 μg/L). Both studies used the ICP-MS methods, and the U.S. reported used 3,699 adults aged 20–64 years. The age distribution of the U.S. [8] was broader than this study and also included both genders. The whole-blood Se reference range in Belgium [9] (P_2.5_–P_97.5_ 83.2–148 μg/L) was similar to our study; Queensland [10] (P_2.5_–P_97.5_ 122–171 μg/L) was higher, and Serbia [13] (P_2.5_–P_97.5_ 52.9–157 μg/L) had lower whole-blood Se distribution than this study.

The countries or regions mentioned above used 95% CI to establish the reference interval. There were also some other description methods for the proposed reference range. P_10_–P_90_ whole-blood Se (65.0–113.8 μg/L) was reported in Southern Brazil [16], which was much lower than in our study (P_10_–P_90_ 95.57–150.21 μg/L). P_5_–P_95_ of whole-blood Se was 105–186 μg/L in Italians [15], which was similar to our current result (P_5_–P_95_ 97.89–157.89 μg/L). Few Asian countries have reported Se levels detected by whole blood. However, several studies in some regions of China obtained a quite different whole-blood Se distribution range. It was reported that age and geographic factors might also affect the whole-blood Se concentration [34]. The reference range of Hunan [23] (P_5_–P_95_ 167–302 μg/L) and Chengde [22] (P_2.5_–P_97.5_ 115.17–206.72 μg/L) for women was higher than this study (P_5_–P_95_ 97.89–157.89 μg/L), but for Shaanxi [21] (P_25_–P_75_ 62.62–84.04 μg/L) it was lower than in this study (P_25_–P_75_ 108.59–137.19 μg/L).

It has been reported that gender difference also affects the concentration of blood Se [35]. Some countries have analyzed gender differences while establishing their own reference ranges of Se. Belgium [9] (women 91.7 μg/L; man 96.8 μg/L), Korea [11] (women 110.06 μg/L; men 117.45 μg/L), and the USA [8] (women 185.6 μg/L; man 190.7 μg/L) reported that women’s Se levels were lower than men’s. Some countries had similar values for both man and women [13,14,15]. However, the Se levels were higher in women than men in Southern Brazil (women 85.4 μg/L; men 81.7 μg/L) [16]. Because Se levels might differ between men and women, it is necessary to establish reference ranges specifically for women. More studies need to be carried out to detect the differences of blood Se distribution between different genders and age groups.

The proposed reference range of plasma Se in this study was used to evaluate the Se nutritional status of a representative sample of 1950 women of child-bearing age. There were 24.95% in this population with a plasma Se level lower than the P_2.5_ limit of the reference range and 5.08% higher than the upper limit of the reference range in this study. We found no significant differences between different age groups. As shown in Table 4, the Se level was the highest in the eastern region, followed by the midlands, and the lowest in the western; the difference was statistically significant. The concentration of plasma Se in the urban population was higher than that in the rural population. Compared with the east and the midlands, the child-bearing-aged women in the western region tended to have a lower plasma Se level and a higher proportion less than the P_2.5_ cut-off value of reference range, while a similar situation was also found in rural rather than in urban areas. Again, that might have demonstrated the effect of geographical factor on the Se nutritional status of the population.

There were also some limitations in this study. Firstly, the absorption and metabolism of Se might be affected by other elements, but the current results were not adjusted by other elements. Secondly, we only reported the preliminary distribution of plasma Se in the total 1950 child-bearing-aged women; the specific influencing factors remain to be further analyzed.

## 5. Conclusions

There were some strengths in this study. Firstly, strict inclusion criteria were used to establish the reference range of blood Se distribution of healthy Chinese women of childbearing age. The sample size achieved the requirements of similar studies and was more age specific, and the results are well consistent with some other countries and regions. Secondly, the reference range of plasma Se in this study was applied to the assessment of Se status in a national representative sample in the latest nutrition monitoring to preliminarily understand the distribution of plasma Se in the population.

In conclusion, this study provided a reference range for Se in plasma and whole blood of Chinese women of child-bearing age (18–44 years). A considerable proportion (24.05%) of women of childbearing age in China have plasma Se levels lower than the low value of 95% CI obtained in this study, and their health effects need to be further studied.

## Figures and Tables

**Table 1:** General characteristics of enrolled healthy women of childbearing age.

Parameters	Geom. Mean	Median	P_25_	P_75_
Age (years)	28.6	29.0	25.0	35.0
Height (cm)	156.9	157.2	153.3	161.1
Weight (kg)	51.8	51.6	48.6	55.1
BMI (kg/m^2^)	21.0	21.2	19.9	22.3
TC (mmol/L)	4.0	4.1	3.7	4.5
TG (mmol/L)	0.7	0.7	0.6	1.0
LDL (mmol/L)	2.2	2.3	2.0	2.6
HDL (mmol/L)	1.4	1.4	1.2	1.6
UA (umol/L)	234.8	242.3	205.0	279.2
SBP (mmHg)	114.2	114.0	108.3	120.7
DBP (mmHg)	70.9	70.3	66.7	76.0
FG (mmol/L)	4.9	4.9	4.6	5.1
HbA1c (%)	4.8	4.8	4.4	5.2
Hb (g/L)	135.8	138	129	144
Heart Rate (t/min)	77.5	76.3	71.7	83.0

BMI—body mass index; TC—total cholesterol; TG—triglyceride; LDL—low-density lipoprotein; HDL—high-density lipoprotein; UA—uric acid; SBP—systolic blood pressure; DBP—diastolic blood pressure; FG—fasting glucose; HbA1c—hemoglobin A1c; Hb—hemalbumin.

**Table 2:** Plasma and whole-blood Se levels (μg/L) in relation to different variables.

Variables	N	Plasma (μg/L)				Whole Blood (μg/L)			
		Geom. Mean	P_2.5_	P_97.5_	*p*	P_50_	P_2.5_	P_97.5_	*p*
Total	187	100.94	73.81	140.75		121.05	81.06	164.72	
Age Group (Years)					0.069				0.355
18–25	72	104.59	75.68	144.83		123.91	84.88	166.20	
25–35	70	98.31	75.87	142.06		118.28	74.80	166.14	
35–45	45	100.46	65.55	136.95		120.88	85.36	159.30	
Area					0.068				0.001
Eastern	65	103.92	74.30	144.91		128.79	85.51	181.42	
Midlands	93	100.46	74.89	140.28		118.45 ^b^	82.60	170.11	
Western	29	91.72	69.33	140.20		114.62 ^b^	80.43	167.62	
Residence region					0.000				0.403
Urban	83	106.00	77.24	140.49		122.69	84.32	164.37	
Rural	104	94.94 ^a^	71.22	142.66		119.76	78.46	164.97	

^a^ Compared with urban, *p* < 0.05; ^b^ compared with eastern, *p* < 0.05.

**Table 3:** Reference range of plasma/serum and whole blood Se in various countries or regions.

Years	Regions	Age (Years)	Gender	Sample Size	Mean/Median	Parameter	Reference Range of Se (μg/L)	Blood Status
	China (this study) *	18–45	Female	187	100.94 ^a^	P_2.5_–P_97.5_	73.81–140.75	Plasma
2021	Belgium [9] *	18–70	Female	202	91.7 ^b^	P_2.5_–P_97.5_	61.6–122	Plasma
2021	Queensland [10] *	19–73	Female	61	123 ^a^	P_2.5_–P_97.5_	98–160	Plasma
2019	Serbian [13] *	18–40	Adult	149	61.5 ^a^	P_2.5_–P_97.5_	37.4–97.5	Serum
2017	Korea [11] *	12–78	Male and Female	258	111.37 ^a^	P_2.5_–P_97.5_	79.07–166.46	Serum
2006	Taiwan [14] ^#^	>15	Adult	2755	110.9 ^a^	min–max	40.5–186	Serum
1996	Japan [12] ^#^	23–51	Adult	51	117.4 ^a^	min–max	76.9–149.4	Plasma
2007	Beijing [19] ^&^	15–84	Female	273	78.85 ^a^	min–max	35.2–160.4	Serum
2009	Jinan [20] ^#^	18–60	Female	288	60.77 ^a^	P_2.5_–P_97.5_	35.58–85.96	Serum
	China (this study) *	18–45	Female	187	121.05 ^a^	P_2.5_–P_97.5_	81.06–164.72	Whole Blood
2021	Belgium [9] *	18–70	Adult	380	110 ^b^	P_2.5_–P_97.5_	83.2–148	Whole Blood
2021	Queensland [10] *	18–72	Female	49	140 ^a^	P_2.5_–P_97.5_	122–171	Whole Blood
2019	Serbia [13] *	18–40	Adult	149	80.3 ^a^	P_2.5_–P_97.5_	52.9–157	Whole Blood
2019	Southern Brazil [16] *	>40	Female	512	85.4 ^a^	P_10_–P_90_	65.0–113.8	Whole Blood
2015	USA [8] *	20–64	Adult	3699	193.2 ^b^	P_2.5_–P_97.5_	152.3–251.3	Whole Blood
2011	Italy [15] *	18–89	Female	111	136 ^b^	P_5_–P_95_	105–186	Whole Blood
2020	Shaanxi [21] *	3–79	Female	356	72.96 ^b^	P_25_–P_75_	62.62–84.04	Whole Blood
2014	Chengde [22] *	6–60	Female	286	157.96 ^b^	P_2.5_–P_97.5_	115.17–206.72	Whole Blood
2015	Hunan [23] *	20–79	Female	513	221 ^b^	P_5_–P_95_	167–302	Whole Blood

* Detected by ICP-MS; ^#^ detected by ASS; ^&^ detected by electron capture detector (ECD); ^a^ mean; ^b^ median.

**Table 4:** Plasma Se distribution (μg/L) in representative child-bearing-aged women of China.

			Se Concentration Range (μg/L)						
Variables	N	Geom. Mean	<73.81		73.81–140.75		>140.75		*p*
			No.	Percentage (%)	No.	Percentage (%)	No.	Percentage (%)	
Total	1950	87.69	469	24.05	1382	70.87	99	5.08	
Age Group									0.266
18–25 years	614	86.25	148	24.10	444	72.31	22	3.58	
25–35 years	672	88.34	154	22.92	480	71.43	38	5.65	
35–45 years	664	88.39	167	25.15	458	68.98	39	5.87	
Area									<0.05
Eastern	652	94.24	88	13.50 ^d^	528	80.98	36	5.52	
Midlands	680	90.02 ^a^	140	20.59 ^d^	499	73.38 ^f^	41	6.03	
Western	618	78.97 ^a,b^	241	39.00	355	57.44 ^f^	22	3.56	
Residences									<0.05
Urban	788	90.92	147	18.65	605	76.78 ^g^	36	4.57	
Rural	1162	85.56 ^c^	322	27.71 ^e^	777	66.87	63	5.42	

^a^ Compared with east, *p* < 0.05; ^b^ compared with midlands, *p* < 0.05; ^c^ compared with urban, *p* < 0.05; ^d^ compared with western, the difference is significant; ^e^ compared with urban, the difference is significant; ^f^ compared with eastern, the difference is significant; ^g^ compared with rural, the difference is significant.

## Data Availability

Not applicable.
